# Differential activation of Ca^2+^ influx channels modulate stem cell potency, their proliferation/viability and tissue regeneration

**DOI:** 10.1038/s41536-021-00180-w

**Published:** 2021-10-20

**Authors:** Naseem Ahamad, Yuyang Sun, Viviane Nascimento Da Conceicao, Caroline R. D. Xavier Paul Ezhilan, Mohan Natarajan, Brij B. Singh

**Affiliations:** 1 Department of Periodontics, School of Dentistry, University of Texas Health San Antonio, San Antonio, TX 78229 USA; 2 Department of Pathology and Laboratory Medicine, School of Medicine, University of Texas Health San Antonio, San Antonio, TX 78229 USA

**Keywords:** Mesenchymal stem cells, Metabolic syndrome

## Abstract

Stem cells have indefinite self-renewable capability; however, factors that modulate their pluripotency/function are not fully identified. Here we show that store-dependent Ca^2+^ entry is essential for modulating the function of bone marrow-derived mesenchymal stem cells (MSCs). Increasing external Ca^2+^ modulated cell cycle progression that was critical for MSCs survival. Additionally, Ca^2+^ was critical for stem proliferation, its differentiation, and maintaining stem cell potential. Ca^2+^ channel characterization, including gene silencing, showed two distinct Ca^2+^ entry channels (through Orai1/TRPC1 or via Orai3) that differentially regulate the proliferation and viability of MSCs. Importantly, NFκB translocation, but not JNK/ERK into the nucleus, was observed upon store depletion, which was blocked by the addition of Ca^2+^ channel inhibitors. Radiation lead to a decrease in saliva secretion, decrease in acinar cell number, and enlarged ducts were observed, which were restored by the transplantation of stem cells that were propagated in higher Ca^2+^. Finally radiation showed a decrese in TRPC1 expression along with a decrese in AQP5, which was again restored upon MSC tranplantation. Together these results suggest that Ca^2+^ entry is essential for stem cell function that could be critical for regenerative medicine.

## Introduction

Stem cells are unique as they possess the indefinite self-renewable capability that can be differentiated into all types of cell lineages, making them unique and ideal candidates for organ development or tissue therapy^1,2^. Several types of stem cells have been identified that are either according to their origin (embryonic, germinal, and somatic stem cells), or are classified based on their differentiating potential (totipotent, pluripotent, and multipotent cell types)^3^. Importantly, stem cells are located in particular anatomic regions that provide the microenvironment or niche needed for their self‐renewal, differentiation, and maintenance. The mammalian bone marrow is the largest reservoir that contains various stem cells such as: hematopoietic stem cells (HSCs), multipotent adult progenitor stem cells (MAPCs), and mesenchymal stem cells (MSCs)^4–7^. Importantly, demand for tissue reconstruction especially for the regeneration of damaged tissues. Due to disease or aging has been expanding^8^; thus, there is a critical need to understand the factors that could modulate their stemness/proliferation.

MSCs are perhaps the most important non-embryonic stem cells as they are quiescent, can self‐renew, and be differentiated into multiple linages^7^. Although MSCs are able to maintain their stemness, it is not fully understood how steady‐state function and homeostatic responses are regulated. Ca^2+^ is a ubiquitous intracellular messenger, which plays a key role in cellular processes such as cell signaling, cell growth/proliferation, protein secretion, exocytosis, and even cell death and apoptosis^9–11^. Stem cells utilize many factors and signaling mechanisms in which Ca^2+^ channels, and Ca^2+^ signaling play an essential role in maintaining the self-renewal and differentiation process^9^. Although various Ca^2+^‐permeable channels such as voltage‐operated channels, receptor‐operated channels, second messenger‐operated channels, and store‐operated Ca^2+^ entry (SOCE) channels are present in the plasma membrane, which are critical for intracellular Ca^2+^ entry in stem cells^12–17^. Activation of G-protein coupled receptors generates IP_3_ that upon binding to the IP_3_Rs in the ER leads to ER Ca^2+^ release and potentiates store-operated Ca^2+^ entry (SOCE) mechanism^18,19^. Mouse embryonic stem cells (mESCs) need Ca^2+^ influx from the extracellular space that is required for its proliferation and activation of Sox-2, Klf-4, and Nanog^20^, but the identity of the Ca^2+^ entry channel is not known. Importantly the bone also harbors high Ca^2+^ concentration (up to 40 mM)^20,21^, which could make them more sensitive to Ca^2+^ fluctuations. Moreover, depending on Ca^2+^ signaling system, a Ca^2+^ signal may lead to proliferation or cell death, suggesting a tight regulation of Ca^2+^ is required for stem cells function.

Two major Ca^2+^ channel families, Orai’s and transient receptor potential canonical (TRPCs) have been identified^12^, but their expression and individual role in stem cell function is not yet identified. Importantly, both TRPCs and Orai’s are regulated by Stromal interaction molecule 1 (STIM1) that is present in the ER and functions as an ER Ca^2+^ sensor^12^. Decrease in ER Ca^2+^ concentration is sensed via an N‐terminal Ca^2+^‐binding EF‐hand motif present on STIM1 and begins to oligomerize in the ER lumen, followed by its translocation/rearrangement to the plasma membrane. The C‐terminal region of STIM1 gates Orai/TRPC channels and stimulates Ca^2+^ entry that could be important for cell fate decisions that are required during stem cells development and function. Ca^2+^ entry through SOCC is critical for the activation of calmodulin-dependent protein kinase II (CAMK-II), and CAMK-II is shown to regulate the regeneration of hematopoietic stem and progenitor cells^22^. Similarly, Ca^2+^ entry is also critical for switching from quiescence to cell cycle activation and Ca^2+^-dependent pathways are shown to modulate stem cell divisions^23^. Suppressing the intracellular Ca^2+^ levels has been shown to prolong cell division interval in hematopoietic stem cells^23,24^. In contrast, low Ca^2+^ has been shown to inhibit calpain proteases, thereby improving stem cell maintenance in vitro^25^, suggesting that a tight regulation of Ca^2+^ is essential for maintaining stem cells function. However, it remains poorly understood as to how Ca^2+^ pathways through various Ca^2+^ channels modulate stem self‐renewal/function. The data presented in this manuscript establish the molecular identity of the calcium channel in MSCs and also show their role in modulating stem cell function

## Results

### Ca^2+^ entry in MSCs is essential for its cell viability, proliferation, and stem cell potential

**Fig. 1:**
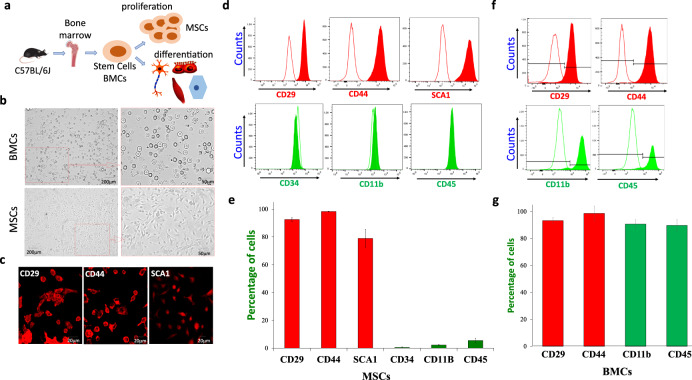
Isolation and characterization of mesenchymal stem cells. **a** Schematic showing isolation of mouse bone marrow cells (BMCs) and its properties of self-renewal and differentiation of MSCs. **b** Representative microscopic images displaying isolated bone marrow cells that show circular and round-shaped morphology of BMCs; whereas, adhered MSCs show fibroblast or eye-shaped morphology. **c** Immunofluorescence images of MSCs showing the expression of MSC-positive markers (CD29, CD44, and SCA1). **d** Immunophenotypic characterization of MSCs using flow cytometer showing the absolute counts of MSC positive markers such as CD29, CD44, and SCA1, as well as expression of MSC-negative (CD34, CD45, and CD11b). Isotype controls for each antibody were used as controls and FITC/rhodamine-conjugated secondary antibodies were used for their detection. **e** Bar graph (quantification from four independent experiments (expressed as mean ± standard error)) showing the percentage of cells expressing the MSC-positive and negative markers respectively. **f**, **g** Immunophenotypic characterization of BMCs using flow cytometer showing expression various markers (CD29, CD44, CD45, and CD11b). Bar graph expressed as mean ± standard error shows quantification of respective markers from three independent experiments.

Bone marrow-derived mesenchymal stem cells (MSCs) were isolated from the central cavity of 1-month-old C57BL/6J mice. Long bones, the femur, and the tibia were cleaned and flushed using saline, and cells obtained were seeded at the density of 1 × 10^6^ cells/cm^2^ in T-75 tissue culture flasks for various experimentation (Fig. 1a). Although the bone marrow contains different types of cells, the MSCs were selected using differential media and characterized further. Unlike the bone marrow cells (BMCs) that maintained the round phenotype, the MSCs isolated were able to adhere to the surface and showed an elongated spindle-shaped morphology within 2 h of incubation (Fig. 1b). Importantly, the expression of stem cell markers (CD29, CD44, and Sca-1) was also observed in MSCs (Fig. 1c). To have more conclusive evidence we also used flow cytometry and expression of both positive and negative markers for stem cells (MSCs) and BMCs were evaluated. As shown here, we found that MSCs showed a differential expression pattern for these stem cell markers. Control MSCs showed expression for all the positive markers: CD29 (92.55% ± 1.56%), CD44 (98.33% ± 0.23%), Sca-1 (78.95% ± 5.50%). Similarly, a significant decrease in all negative markers tested (CD34 (0.55% ± 0.47%), CD11b (1.53% ± 0.44%), and CD45 (2.20% ± 1.66%)) was observed (Fig. 1d, e and Supplementary Fig. 1a, b). In contrast, BMCs showed the expression of both stem cells (CD29, CD44) and immune cells (CD11b, CD45) markers (Fig. 1f, g), and no decrease in the expression of immune cell markers was observed in BMCs, when compared with MSCs.

To further establish the properties of stem cells, we examined the MSCs differentiation potential. MSCs were cultured under adipogenic and osteogenic conditions that allow them to be differentiated into the mesodermal lineages. Using standard Oil red staining for adipocyte and Von Kossa staining for osteocyte, we found that control undifferentiated cells did not show any morphological and cytoplasmic changes and were negative for oil red staining. In contrast, MSCs grown in adipogenic medium for 8–10 days showed cytoplasmic lipid droplets and oil red staining was prominently observed in the majority of cells (Supplementary Fig. 1c). Similarly, MSCs grown in osteogenic medium for 10–12 days showed dark brown-black mineral disposition (Supplementary Fig. 1d) confirming that these cells are able to differentiate into various linages. Together, these results establish that the cultured MSCs demonstrate a spindle-shaped morphology along with increased expression of positive surface markers, and possess the differentiation potential, thereby fulfill the stem cell criteria.

**Fig. 2:**
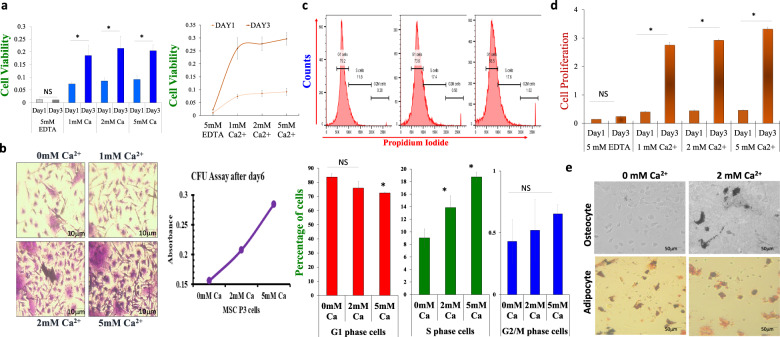
Role of calcium on MSCs viability and cell proliferation. **a** MTT assays were performed to investigate cell viability is shown as bar graph (expressed as mean ± standard error) or Liner plot. MSCs cells were treated with 5 mM EDTA (for 0 mM Ca^2+^) and increasing doses of calcium for 1 and 3 days and MTT assays were performed. **b** Colony-forming unit (CFU) assay shows the stem cell potential after incubation with different calcium concentrations as labeled in the figure. CFU microscopic images and line-graph show the amount of stem cell potential with increasing Ca^2+^ concentrations. **c** Cell-cycle analysis was performed to study the different phases of the cell cycle after varying calcium treatment. The histogram and quantification of the data (shown as bar graph expressed as mean ± standard error) show the increment in the cell growth phase, i.e., S phase, was increased with increasing Ca^2+^ concentrations **d** BrdU assay was performed to study the cell proliferation of MSCs after treatment (1 and 3 days) with 5 mM EDTA and different doses of calcium, which is expressed as mean ± standard error from four independent experiments. **e** Microscopic images showing MSCs differentiation into adipocytes and osteocytes lineages in various calcium concentrations. Mineral deposition (Van Kossa staining) and lipid droplets (oil red staining) was used to evaluate osteocyte and adipocyte differentiation respectively. NS indicates data not significant and *p ≤ 0.001 (Student’s *t*-test).

Next, we evaluated the factors that are essential for stem cell function. Importantly, Ca^2+^ is been shown to play a vital role in modulating opposing cellular function such as cell proliferation and cell death^17,26^. Thus, initially we used MTT and BrdU assays to establish the role of Ca^2+^ in stem cell viability and in the proliferation of MSCs. MSCs were seeded in 96-well plates and treated with increasing external Ca^2+^ concentrations (0 mM Ca^2+^+5 mM EDTA, 1 mM Ca^2+^, 2 mM Ca^2+^ and 5 mM Ca^2+^) for 1 and 3 days, and cell viability was measured. Importantly, increasing external Ca^2+^ levels showed an increase in cell viability; however, cells that were devoid of external Ca^2+^ showed a significant loss of MSCs (Fig. 2a). On the other hand, cells treated with higher external Ca^2+^ showed a significant increase in cell survival at 3 days post treatment, when compared with cells at day 1 (Fig. 2a). Colony-forming units (CFU) assay were also performed to analyze the role of external Ca^2+^ in their stem cell potential. MSCs were seeded and treated with different doses of Ca^2+^ (0 mM Ca^2+^, 2 mM Ca^2+^, and 5 mM Ca^2+^) for 24 h, and CV absorbance was analyzed. Interestingly, a dose-dependent increase in stem cell potential was observed in the presence of external Ca^2+^ and absorbance values were gradually increased even at 5 mM Ca^2+^-treated cell (Fig. 2b). Again, cells growing in 2 mM Ca^2+^ showed a −2-fold increase in CV absorbance compared to 0 mM Ca^2+^-treated cells (Fig. 2b).

To further identify how Ca^2+^-treated cells showed increased cell survival and exhibited enhanced stem potency, cell cycle was also evaluated in these conditions. Again, MSCs were treated with different Ca^2+^ concentrations (0 mM Ca^2+^, 2 mM Ca^2+^, and 5 mM Ca^2+^) and cell cycle status was observed using Flow cytometry. Interestingly, increasing external Ca^2+^ lead to a decrease in the percentage of G1 phase cells; whereas, the percentage of S phase cells was significantly increased (9.03 ± 1.43%, 13.87 ± 1.87%, 18.73 ± 0.78% after 0 mM Ca^2+^, 2 mM Ca^2+^, and 5 mM Ca^2+^ treatment) (Fig. 2c). Consistent with these results, cells treated with 5 mM EDTA showed no significant increase in cell proliferation at day3, whereas increasing Ca^2+^ concentration (1–5 mM) showed significantly higher cell proliferation at day 3, when compared to day 1, respectively, (Fig. 2d). Moreover, flow cytometry further showed that PCNA (proliferating cell nuclear antigen) levels were increased in MSCs that were treated with higher doses of Ca^2+^ (Supplementary Fig. 2a–c, f). Data also showed that cells treated with high Ca^2+^ showed a gradual increase in intracellular Ca^2+^ levels, (Supplementary Fig. 2h). Interestingly, MSCs grown in osteogenic medium supplemented with 2 mM Ca^2+^ for 10–12 days showed dark brown-black mineral disposition, which were absent in media devoid of external Ca^2+^ (Fig. 2e). Surprisingly, differentiation of MSCs into adipocytes was still observed in media devoid of external Ca^2+^ and MSCs supplemented with 2 mM Ca^2+^ also showed similar adipocytes differentiation (Fig. 2e). Together these results show that Ca^2+^ is crucial for stem cell viability and for its proliferation and Ca^2+^ has a differential role in the differentiation of MSCs into various cell linages.

### Characterization of Ca^2+^ channels responsible for cell proliferation in stem cells

**Fig. 3:**
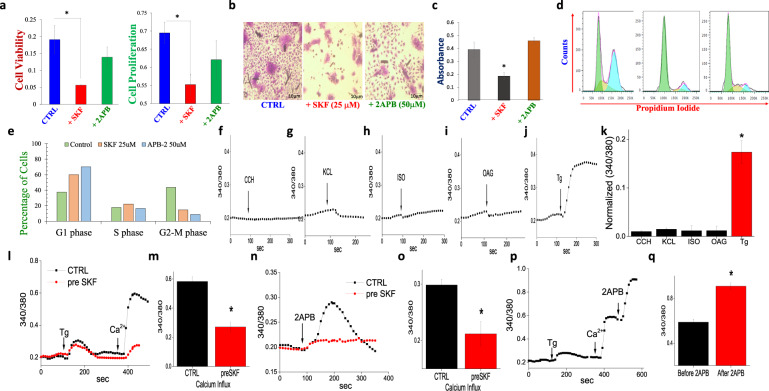
Characterization of SOCE channel of MSCs. **a** MTT and BrdU assay were performed to investigate calcium’s proliferative effect. MSCs cells were treated with SKF 96365 and 2-APB for 24 h. Bar graphs depict average ± SE in 3–5 separate experiments, *p ≤ 0.001 (Student’s *t*-test). **b** Microscopic image for the colony-forming unit (CFU) assay shows the stem cell potential after the treatment of calcium channel blockers. **c** Graph shows decreased stem cell potential (crystal violet staining, CV) with the addition of calcium channel blockers, the data show are mean ± standard error from four independent experiments performed in duplicate. **d**, **e** Cell cycle assay was done to evaluate cell cycle phases after the treatment of calcium channel blockers. The histogram and graph show (mean ± standard error) the increment of G1 phase cells and decline the percentage of the G2/M phase with channel blockers. **f**–**j** Individual traces showing changes in cytosolic calcium levels upon addition of KCL (100 mM), OAG (50 µM), Isoproterenol (100 µM), Carbachol (50 µM) and Thapsigargin (2 μM) in MSC cells. **k** shows quantification (as bar graph mean ± standard error) of relative calcium entry in various stimulation from 40 to 60 individual cells. **l** Tg evoked Ca^2+^ release from the ER, and Ca^2+^ entry in control MSC cells and MSCs pretreated 50 µM SKF 96365. **m** Quantification (as bar graph expressed as mean ± standard error) of relative calcium entry in control and SKF-treated cells (data shown are average of 50–70 cells). **n** Representative traces showing 2-APB (100 µM) evoked [Ca^2+^]_i_ changes in MSCs under various conditions as labeled in the figure. Pre-application of SKF 96365 (50 µM) abolished 2-APB induced changes in [Ca^2+^]i. **p** addition of 100 µM 2-APB potentiated Tg evoked Ca^2+^ influx in MSC cells. **o**, **q** Quantification (as bar graph) of relative calcium entry in various conditions from 50 to 90 individual cells. *p ≤ 0.001 (Student’s *t*-test).

Data presented thus far show that Ca^2+^ entry is essential for stem cell function; however, the ion channel essential for Ca^2+^ entry is not yet defined. To identify the molecular identity of the Ca^2+^ channel, we initially used different Ca^2+^ channel inhibitors. Interestingly, when cells were treated with non-specific Ca^2+^ entry channel blockers SKF 96365 (25 μm), a significant decrease in cell viability was observed (Fig. 3a). In contrast addition of another SOCE inhibitor 2-APB (50 μm) (Fig. 3a) or the voltage-gated Ca^2+^ channel inhibitor (Nifedipine, data not shown), failed to show any loss of cell viability. These results indicate that Ca^2+^ entry via the SOCE channels may be responsible for cell viability. Furthermore, we also found that SKF 96365-treated cells showed a 2.12-fold decrease in cell proliferation compared to untreated control cells, and 2-APB-treated cells showed only a partial 1.31-fold decrease in cell proliferation than control (Fig. 3a). Importantly, the addition of Ca^2+^ channel inhibitors also showed a decrease in PCNA levels, but intracellular Ca^2+^ level was only decreased in SKF 96365-treated cells (Supplementary Fig. 2d, e, g, i). MSCs were treated with Ca^2+^ channel blockers and stem cell potential was evaluated. Interestingly, SKF 96365-treated cells showed a significant (2.05-fold) decrease in stem cell potential (as measured by crystal violet (CV) absorbance). In contrast, 2-APB-treated cells showed a 1.18-fold increase in CV absorbance when compared with control, which indicates that 2-APB somehow potentiates stem cell potential (Fig. 3b, c). However, the addition of Ca^2+^ channel inhibitors showed an increase in the G1 phase, along with a decrease in the G2/M phase, without any change in the S phase (Fig. 3d, e), suggesting that multiple Ca^2+^ entry channels might regulate differential function in MSCs.

To further characterize Ca^2+^ entry mechanism that is critical for stem cell function, we used different agonist and intracellular Ca^2+^ concentration was evaluated. Carbachol, which is a muscarinic/nicotinic receptor agonist and stimulates Ca^2+^ release through co-operation between IP_3_Rs/RyRs failed to show any increase in intracellular Ca^2+^ levels (Fig. 3f, k). Similarly, KCl that activates the voltage-gated channels also showed no increase in intracellular Ca^2+^ spike, suggesting that voltage-gated channels are not involved in increasing cytoplasmic Ca^2+^ levels in MSCs (Fig. 3g, k). Isoproterenol (ISO), an α1/β-adrenergic receptor agonist that increases cAMP and sensitize the IP_3_-mediated intracellular Ca^2+^ in the cells, also showed no evoked Ca^2+^ spike (Fig. 3h, j, k). Similarly, 1-oleoyl-2-acetyl-*sn*-glycerol (OAG), which is a membrane-permeant and a synthetic analog of endogenous second messenger diacylglycerol (DAG) also failed to show any increase in Ca^2+^ levels (Fig. 3i, k). Interestingly, OAG not only activates TRPC3/6 channels, but also stimulates the T-type voltage-gated channels, again suggesting that neither voltage-gated channels nor some of the TRPC channels are critical for Ca^2+^ entry in MSCs. Interestingly, when MSCs were treated with Thapsigargin (Tg), a potent inhibitor of sarco-endoplasmic reticulum Ca^2+^-ATPases that causes ER depletion, a rapid increase in cytosolic Ca^2+^ levels were observed (Fig. 3j, k). These data further strengthen that Ca^2+^ entry required for MSCs function was mediated through the store-operated Ca^2+^ entry (SOCE) mechanism.

To further establish the importance of SOCE, MSCs were pretreated with SKF 96365 (a non-selective Ca^2+^ channel blocker), which again showed a significant decrease in Ca^2+^ entry, without altering Ca^2+^ release from the ER stores (Fig. 3l, m). In contrast, the addition of 2-APB, showed a significant increase in Ca^2+^ entry in MSCs, which was though inhibited by the pre-treatment of SKF 96365 (Fig. 3n, o). Importantly, 2-APB has been shown to increase STIM1-Orai3 channel conductance and activates Ca^2+^ entry at low concentrations; thus, we immediately added 2-APB in cells that were already stimulated with Tg and showed Ca^2+^ entry. The addition of 2-APB further potentiated Ca^2+^ entry, which was even higher than what is observed after store depletion induced SOCE (Fig. 3p, q). Together these results suggest that distinct store-operated Ca^2+^ entry channels are present (via Orai1/TRPC1 or Orai3 channels) could induce Ca^2+^ entry in MSCs.

### Distinct Ca^2+^ entry channel modulates cell proliferation and viability in stem cells

**Fig. 4:**
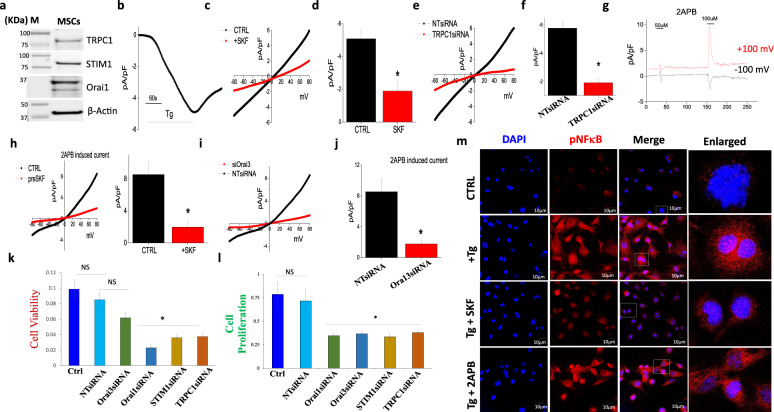
MSCs cell viability and cell proliferation after treatment of calcium channel blockers. **a** Western blot showing expression of calcium channel proteins in MSCs. **b** Application of 2 µM thapsigargin (Tg) in bath solution induced an inward Ca^2+^ current which is shown at −80 mV in MSC cells. **c** Respective IV curves of Ca^2+^ currents and its quantitation (5–8 recordings) of current intensity at −80 mV are shown in **d**. **e** IV curves of Ca^2+^ currents in control non-targeted siRNA (NTsiRNA) and TRPC1siRNA-treated cells. Quantitation (7–9 recordings) of current intensity in individual conditions at −80 mV are shown in **f**. * Indicate significance (*p* < 0.05). **g** 2APB-induced Ca^2+^ currents at −100 and +100 mV. **h** Respective IV curves and its quantitation (6–8 recordings) of 2APB-induced current intensity in control and SKF-treated cells. **i** IV curves of 2APB-induced Ca^2+^ currents in control non-targeted scrambled siRNA (NTsiRNA) and Orai3siRNA-treated cells. Quantitation (5–6 recordings) of current intensity in individual conditions at −80 mV are shown in **j**. * Indicate significance (*p* < 0.05). **k**, **l** MSCs were silenced with individual siRNAs (STIM1, TRPC1, Orai1, and Orai3), and cell viability and proliferation were studied. NS indicates non-significant, whereas, *Significance *p* ≤ 0.001 (Student’s *t*-test) from four independent experiments performed in duplicate, the data shown are mean ± standard error. **m** Immunofluorescence of pNFkB expression in control (CTRL, after Tg treatment (+Tg) or, SKF-96365, and 2-APB pre-treated cells followed by the addition of Tg).

To investigate whether MSCs express these individual Ca^2+^ channels, western blotting was performed, which showed that stem cells express Orai1, STIM1, and TRPC1 proteins (Fig. 4a). Although SOCE channels have been shown to be mediated via Orai1/STIM1 or TRPC1/STIM1, both these channels have distinct electrophysiological characteristics. Thus, electrophysiological measurements were performed, which showed that the addition of Tg, induced a non-selective current (Fig. 4b–d). Importantly, the properties of the SOCE channel were similar to that has been observed with TRPC1, which are non-selctive^27^ that is distinct from what has been previously shown for Orai1, (inward rectifying currents)^28^. Importantly, the addition of SKF 96365 significantly inhibited the currents without changing the channel properties (reversal potential was still at 0 mM and only the amplitude of the current was decreased) (Fig. 4c, d). In addition, silencing of TRPC1 in MSCs not only showed a decrease in TRPC1 protein levels (Supplementary Fig. 2j) but, also showed a decrease in Tg-induced activation of the calcium current (Fig. 4e, f). The addition of 2APB again induced a non-selective calcium current, which was although inhibited by the addition of SKF-96365 (Fig. 4g–i). Next, we evaluated if indeed 2APB-induced currents are dependent on Orai3. Interestingly silencing of Orai3 not only decreased Orai3 protein levels (Supplementary Fig. 2j) but, also showed a decrease in 2APB-induced calcium currents without altering its properties (Fig. 4i, j).

To further confirm the functional role of these individual calcium entry channel(s) in stem cell function, MSCs were seeded in 96-well plates, and STIM1, TRPC1, Orai1, and Orai3 genes were silenced individually that showed a decrease in individual protein levels, without altering the expression of actin (Supplementary Fig. 2j). Interestingly, silencing of either TRPC1 or STIM1, or Orai1 showed a significant decrease in cell viability, whereas Orai3-silenced cells showed no significant decrease in cell viability even after 24 h (Fig. 4k). No decrease in cell viability was observed in cells that were transfected with non-targeted siRNA (NTsiRNA), when compared with control (Ctrl) untreated cells, On the other hand, cell proliferation was dependent on Ca^2+^ entry and STIM1, TRPC1, Orai3, or Orai1-silenced cells showed a significant decrease in cell proliferation (Fig. 4l). To further investigate the functional role of Ca^2+^ entry channels, cells were treated with Tg, SKF 96365, and 2-APB, and the intracellular localization of pNFκB was studied in the MSCs by immunofluorescence. Results showed that control cells showed more cytoplasmic expression and no nuclear localization of pNFκB was observed (Fig. 4m and Supplementary Fig. 3b). In contrast, Tg-treated cells showed an increase in the nuclear translocation of pNFκB when compared to untreated control cells, which were blocked in cells that were pretreated with either SKF 96365 or 2-APB (Fig. 4m and Supplementary Fig. 3b). In contrast, the nuclear expression of extracellular-signal-regulated kinase (pERK) and c-Jun N-terminal kinase (pJNK) was not altered (Supplementary Fig 3a). Together these results suggest that distinct Ca^2+^ entry channels have individual roles, where SOCE channels (Orai1/3/TRPC1) are important for stem cell proliferation, whereas cell viability is only dependent on Orai1 and TRPC1.

### Restoration of salivary gland function after radiation-induced damage is dependent on transplantation of MSCs

**Fig. 5:**
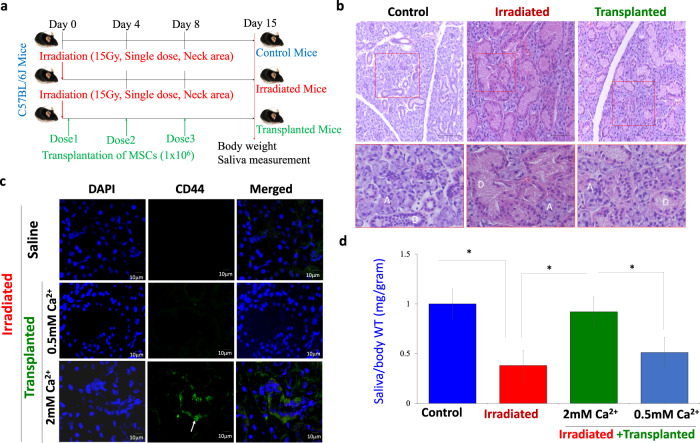
Regeneration of irradiation damaged salivary gland by transplanting MSCs to irradiated mice. **a** Flow diagram showing the schematic for the experimental plan. **b** H&E staining showing glandular morphology of salivary gland in control (mock radiated), irradiated and irradiated **+** transplanted MSCs. Images were taken using ×20 objective and scale bar = 100 μm. A, indicates acinar and D indicates ductal cells. **c** Confocal images showing the presence of stem cell marker (CD44, shown by arrow) in the salivary gland of mice from radiated and MSCs transplanted mice growing in 0.5 mM Ca^2+^ or 2 mM Ca^2+^ after radiation treatment. **d** The bar graph represents saliva secretion in control, irradiated and MSCs transplanted mice post radiation. The data presented are from 8 to 10 mice in each group expressed as mean ± standard error. *p ≤ 0.001 (Student’s *t*-test).

To further establish the importance of Ca^2+^ entry, we next studied whether MSCs that are treated with higher Ca^2+^ concentrations and showed increased proliferation had a role in tissue regeneration. Stem cells have been suggested as a possible mechanism for salivary gland regeneration^29,30^. Thus, mice salivary glands were exposed to a single dose of 15 Gy using a computed tomography (CT) image-guided small animal irradiator, and glandular regeneration by MSCs transplantation was evaluated. As shown in Fig. 5a, individual mice were randomly segregated into 3 groups, group1 was used as control (non-irradiated and no MSCs transplantation). Group 2 mice were radiated but did not receive MSCs transplantation; whereas group 3 mice not only receive the desired radiation dose but, were followed by 3 doses of MSCs transplantation. Mice were irradiated specifically at the salivary gland area, which was confirmed using CT images (Supplementary Fig 3c). In addition, body weight, food, and water intake were also evaluated in these 3 groups, which was decreased in irradiated mice (Supplementary Fig 3d, e). Importantly, the histological analysis also showed that radiation-damaged salivary gland cells and an increase in ductal cells (both the number of as well as enlarged ducts) were observed in irradiated mice, along with a decrease in the number of acinar cells (Fig. 5b). Transplantation of exogenous MSCs that were growing in 2 mM calcium showed a more preserved salivary gland structure and a greater number of acini were observed in irradiated mice with MSC transplantation, when compared with irradiated mice alone (Fig. 5b). Importantly, immunohistochemical (CD44) analysis of irradiated and transplanted salivary glands groups, further showed that exogenous MSCs that were cultured in higher calcium concentrations were significantly higher when compared with MSCs that were grown in 0.5 mM Ca^2+^ and transplanted in irradiated mice (Fig. 5c and Supplementary Fig. 3f). Moreover, salivary gland tissues in the reconstituted group were morphologically similar to the mock-irradiated control submandibular glands with both ductal and acinar intact structures were observed (Fig. 5b, c). Saliva collection data clearly showed that the amount of saliva collected from irradiated mice was significantly decreased (0.35 mg/body weight) compared to non-irradiated controls (1.02 mg/body weight) (Fig. 5d). Interestingly, mice that were irradiated, but received MSCs growing in 2 mM Ca^2+^ showed a significant restoration in their ability to secrete saliva (0.92 mg/body weight) (Fig. 5d). In contrast, transplantation of stem cells that were cultured in 0.5 mM Ca^2+^ was only partially able to rescue the radiation-induced loss of saliva secretion (0.51 mg/body weight) (Fig. 5d), suggesting that calcium has a positive role in stem cell restorative function.

**Fig. 6:**
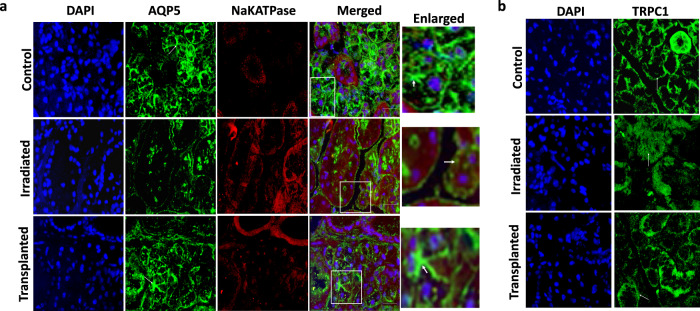
Expression of channel proteins in salivary glands under various conditions. **a** Expression of acinar (AQP5, shown as white arrow) and ductal (Na^+^/K^+^-ATPase) in control (mock radiated), irradiated, and irradiated + transplanted (2 mM Ca^2+^) salivary gland tissue sections and **b** confocal images showing expression of TRPC1 channels in control (mock radiated), irradiated, and irradiated + transplanted salivary gland issue sections. Images were taken using ×40 objective and scale bar = 50 μm.

In addition, expression of Aquaporin 5 (AQP5) protein that forms an exocrine gland-type water channel and transfers water and small solutes across acinar cells was also decreased in irradiated mice (Fig. 6a). Similarly, localization of AQP5, which is present at the liminal end of the acinar cells was also decreased (Fig. 6a). In contrast, both control and transplanted mice showed restoration of AQP5 expression and its localization was again at the luminal end (marked with an arrow), suggesting that the restoration of salivary gland function in transplanted MSCs leads to functional acini (Fig. 6a). Na^+^/K^+^-ATPase, a key enzyme that maintains sodium and potassium homeostasis and expressed in ductal cells was also evaluated. Importantly, an increase in Na^+^/K^+^-ATPase expression was observed in irradiated mice, which was again decreased in transplanted mice (Fig. 6a), again suggesting that transplanted MSCs (growing in 2 mM Ca^2+^ can restore radiation-induced damage to salivary gland cells). Furthermore, expression of TRPC1 that was localized in the plasma membrane (basolateral region) of the acini, was decreased and a diffused TRPC1 staining was observed in irradiated mice (Fig. 6b). Importantly, MSCs transplanted group showed restoration of acini, as well as TRPC1 expression, which was partially restored in the basolateral membrane of the acinar cells (Fig. 6b). Moreover, mice that were followed by MSC transplantation also showed restoration of food and water consumption along with the restoration of the body weight (Supplementary Fig 3d, e). Together these results suggest that MSCs have the potential to restore salivary gland function and thus could be used for tissue regeneration in damaged salivary glands that undergo radiation.

## Discussion

Mammalian bone marrow is considered the largest source of adult stem cells that are not derived from embryonic tissues. A variety of stems cells are present in the bone marrow that includes hematopoietic stem cells (HSCs), mesenchymal stem cells (MSCs), multipotent adult progenitor cells (MAPCs), and even small embryonic-like stem cells (VSELs) have been identified^5,31^. Though MSCs can be isolated, cultured, propagated, and characterized from other tissues, bone marrow still remains the most common and consistently trustworthy source for in vitro studies and thus could be used for regenerative therapy. Although in vitro culture systems could provide a decent number of stem cells that could be used for initial characterization, they are still not sufficient for organ development or for the repair of damaged tissues. In addition, recent research has revealed that stem cells do not remain static and their properties, including surface marker expression, and stem cell potential is decreased as they are passaged^32,33^. Similarly, not only different passage but, growth factors, physiochemical factors, and activation of various transcription factors/genes are also responsible for changing their phenotypes and decreasing their proliferation ability^34^. Thus, factors that could maintain the proliferation of stem cells are needed. Cell proliferation is a complex mechanism that is orchestrated by several proteins; however, most of these pathways are related to Ca^2+^ signaling mechanisms. Normal cells including stem cells require high external Ca^2+^ concentrations to trigger cell proliferation, while the demand for Ca^2+^ entry in tumor cells is much less^17,26^, suggesting that calcium has a beneficial role in modulating cell proliferation that does not lead to cancer. The data presented here show that external Ca^2+^ is essential in maintaining stem cell proliferation. Increasing external Ca^2+^ leads to increased proliferation as well as increased stem cell potential was observed. On the other hand, removing external Ca^2+^ or blocking calcium entry by the addition of Ca^2+^ channel inhibitors was sufficient to inhibit cell growth and its stem cell potency was also inhibited. Thus, understanding the regulation of Ca^2+^ signaling during stem cell proliferation is critical in order to maintain cell physiology as well as its function in tissue regeneration. Ca^2+^ signaling does involve several proteins, in particular, the signaling between the ER and plasma membrane is essential to maintain cytosolic Ca^2+^ levels, which is required for the activation of transcription factors that modulate cell proliferation. Although several Ca^2+^ entry channels are known, the major Ca^2+^ entry mechanism in non-excitable cells is through SOCE^20,35,36^. Our data further show that in MSCs Ca^2+^ entry upon store depletion is via the SOCE channels. Stimulation of cells with other agonists that activates either receptor or voltage-gated Ca^2+^ channels failed to show any increase in cytosolic Ca^2+^ levels. Interestingly, previous studies have shown that SOCE is important in stem cells, but the molecular identity of the Ca^2+^ entry channel in these cells is not yet determined.

Although two distinct Ca^2+^ entry channel (via TRPC1 and Orai1) that are activated upon store depletion are observed, our data clearly show that both of them are important for stem cell function. Interestingly, although the properties of the endogenous calcium entry channel were non-selective that are mainly mediated via TRPC1, loss of Orai1 still showed a decrease in cell proliferation and its viability. Interestingly TRPC1 has been shown to be regulated by Orai1, where Orai1 modulates TRPC1 plasma membrane expression. This explains as to why loss of Orai1 could modulate stem cells function. Interestingly, the silencing of STIM1 also showed a significant decrease in both cell proliferation and its viability. STIM1 has been shown as a Ca^2+^ sensor that is present in the ER and plays a critical role in modulating SOCE^12,13^. Upon store depletion, the EF-hand of STIM1 is able to sense a decrease in ER Ca^2+^ levels, which allows STIM1 to be spatially redistributed in ER–PM junctions where it could interact with both Orai1 and TRPC1 and facilitate SOCE^37,38^. Our results also indicate that Ca^2+^ entry via the SOCE channels, in turn, increases the activation of downstream pathways that involved the translocation of pNFκB from the cytoplasm to the nucleus. NFκB translocation into the nucleus activates transcription of gene networks related to cell survival responses or proliferation. Importantly, the addition of SOCE inhibitor (SKF 96365) significantly inhibited the translocation of pNFκB, which could be the reason that cell proliferation was inhibited. Ca^2+^ transfer between various organelles such as in the ER and mitochondria is needed to avoid excessive or prolonged changes in cytosolic Ca^2+^ levels, which could be detrimental for the cell.

Our data further show that stem cell viability was also dependent on Ca^2+^ entry and although mitochondrial Ca^2+^ was not evaluated, it could be essential for maintaining cell viability. The cell cycle is also essential in maintaining stem cell function and loss of SOCE has been shown to induce cell cycle arrest at the G0/G1 phase. Interestingly, increasing external Ca^2+^ levels allowed stem cells to move from G1 to S phase that could also play a critical role in modulating stem cell functions. Increased proliferation, due to a decrease in the G1 phase is correlated with down-regulation of expression of the cell cycle inhibitor p27kip1 that is known to augment Ca^2 +^ oscillation frequency. Orai3 has been shown to be activated by the addition of 2APB and has been shown to modulate cell proliferation, which is consistent with our data as loss of Orai3 also showed that cell proliferation was inhibited. In contrast, cell viability was not altered upon Orai3 silencing, but was inhibited by either TRPC1, Orai1, or STIM1 silencing, suggesting that differential calcium channels could have a different functions in stem cells. Although our data showed a role of Orai3, some of these actions may occur regardless of intracellular Ca^2+^ involvement as suggested previously, and more research is needed whether removal of calcium could further inhibit cell viability and cell proliferation in Orai3-silenced cells.

Aquaporin 5 plays many biological functions, such as the generation of saliva, tears, and pulmonary secretions^39^. Previous studies have shown that irradiation downregulates AQP5 expression in the submandibular gland, resulting in decreased saliva secretion^40,41^; however, identification of factors that could repair damaged salivary glands is still not fully identified. Adult stem cells are homologous and possess a low risk that could be used as an alternative to generate structure-specific functional organs^42,43^. In this study, we were able to show that MSCs that were populated in higher Ca^2+^ concentrations were able to partially restore salivary gland function in irradiated mice. Infused MSCs were present in salivary glands and H&E staining showed that infusion of MSCs restored glandular morphology that was altered due to irradiation. Although the number of MSCs observed in radiated mice was quite limited, they were sufficient to increase salivary gland function. One of the possible mechanisms could be that they might provide the niche and support the development of endogenous salivary gland stem cells. Alternatively, direct administration of MSCs has been shown to restore blood flow within submandibular gland tissues, which could also assist in the restoration of salivary glands that would lead to increased saliva secretion and future research is needed to fully characterize these stem cells. Nonetheless, this mechanism could be applied for human salivary gland stem cells generation in vitro that could assist in patients undergoing radiation therapy.

## Methods

### Isolation, culture, enrichment of mesenchymal stem cells (MSCs), and gene silencing

Primary MSCs were isolated from the bone marrow of the femur and the humeri of C57BL/6J mice (obtained from Jackson lab, stock number 000664), as described earlier^6^ with certain modifications. Briefly, mice (4–6 weeks old) were used and long bones were dissected in sterilized conditions. The epiphysis of each bone was removed with sterile scissors, and tubes containing the bones were placed in a 15 ml polypropylene culture tube and centrifuged at 10,000 rpm, 5 min at 37 °C. BMCs palette were dissociated by vortexing to make single-cell suspension and 5 ml complete culture medium, [(Dulbecco’s Modified Eagle’s Medium (DMEM, Cat no. D5796, Sigma), 15% heat-inactivated fetal bovine serum, FBS (Cat no. F4135, Sigma) and 1× anti-anti solution (Cat no. 15240-062, Gibco))] was added into the tube. Cells were passed through a 70 μm cell strainer and the single-cell suspension was seeded (1 × 10^6^ cells/cm^2^) in T-75 cell culture flasks and placed at 37 °C in a 5% CO_2_ humidified incubator. Media was replaced with fresh media, and floating cells containing non-adherent, dead cells were removed after 48 h. Afterward, 70% of old media was replaced with fresh media every three days until the adherent cells reached 80% to 90% confluency. Adherent cells were harvested by trypsinization using 0.25% trypsin/1 mM EDTA (Cat no. 25200-056, Gibco) at 37 °C for 2 min, followed by the addition of the complete medium (10 ml). The cell suspension was centrifuged and resuspended in a complete medium and cultured up to three passages, which were used for all the experiments. For silencing stem cells (2 × 10^4^ cells/cm^2^) were seeded in 96-well plates and 10 μl Opti-MEM medium (Cat no. 11058-021, Gibco) containing respective si-RNA and lipofectamine RNAiMAX (Cat no. 13778-150, Invitrogen) was added for 4 h and following 100 μl complete media was added to each well. Then after plates were incubated for 3 days at 37 °C in a 5% CO_2_ humidified incubator. Orai1 (Cat no. s99510, Ambion), Orai3 (Cat no. s114488, Ambion), STIM1 (Cat no. 151019, Ambion), TRPC1 (Cat no. 187432, Ambion) siRNAs were used.

### Immunophenotypic characterization of MSCs and their differentiation

Stem cells were washed with 1×PBS, harvested by trypsinization, and counted by trypan blue assay. One million cells were resuspended in ice-cold neutralizing buffer (10% FBS, I% sodium azide in 1×PBS), centrifuged at 500 × *g*, 5 min, at 4 °C, and discarded supernatant. Cell pellets were resuspended in staining buffer (Cat no. 00-4222-26, Invitrogen), added antibodies in cell suspension from eBioscience according to company instruction, and cells were incubated for 1 h at 4 °C in the dark. Mesenchymal stem cell (MSC)-positive makers [PE-conjugated antibodies CD29 (Cat no. 12-0291-82), CD44 (Cat no. 12-0441-82), Ly-6A/E (Sca-1) (Cat no. 12-5981-82), and MSC-negative makers [FITC-conjugated antibodies CD11b (Cat no. 11-0112-82), CD45 (Cat no. 11 0451-82), CD34 (Cat no. 11-0341-82)]] were used along with their isotype controls for MSC characterization. Cells were centrifuged at 500 × *g*, 5 min at 4 °C to remove the unbound antibody. Cell pellets were vortexed and resuspended in 100 μl staining buffer. About 50,000 cells were analyzed by BD LSR-II using BD FACSDiva 8.0.1 software, and the data were analyzed by FlowJo_v10.6.2 software. For adipocyte and osteocyte differentiation, cells were seeded at the density of 1 × 10^4^ cells/well in 6-well tissue culture plates under various conditions as labeled. Following 48 h, cultured media was removed, and adipocyte differentiation/induction medium [DMEM-LG (Cat no. D6046, Sigma), 2% FBS, 1×-Anti-anti solution, 5 μg/ml insulin (Cat. no. 19278, Sigma), 1 μM Dexamethasone (Cat. no. G9891, Sigma), 50 μM indomethacin (Cat. no. 17378, Sigma) and 500 nM isobutyl methylxanthine, IBMX (Cat. no. 17018, Sigma)] and osteocyte differentiation/induction medium [DMEM-LG, 2% FBS, 1× Anti-anti solution, 50 μM ascorbic acid (Cat. no. A8960, Sigma), 10 nM Dexamethasone, 10 mM β-glycerolphosphate (Cat. no. G9891, Sigma)] were added to the respective plates. The induction medium was changed every three days for two weeks. The formation of oil droplets and adipocytes was evaluated by oil-red O staining; and dark brown, blackish mineral deposition, and osteocytes formation was evaluated by Von Kossa staining. A bright-field microscope was used to capture individual images.

### Cell proliferation and cell-cycle assessment

Cell proliferation was determined by Cell Proliferation ELISA BrdU colorimetric kit (Cat no. 11647229001, Roche). Briefly, stem cells (2 × 10^4^ cells/PBS) were seeded in 96-well plates and treated with different calcium conc. (0 mM Ca^2+^, 2 mM Ca^2+^ and 5 mM Ca^2+^) and calcium channel blockers (25 μm SKF 96365 and 50 μm 2-APB) for 1 and 3 days. After day 1 and day 3, the medium was aspirated from each well. BrdU labeling, fixing, incubation with anti-BrdU antibody, washing, and substrate reaction steps were performed according to kit instructions. The absorbance was measured in a microplate reader (Synergy™ HTX Multi-Mode Microplate Reader) at 370 and 492 nm. At 80% confluency, the medium was aspirated from each dish, and cells were treated with different calcium conc. (0 mM Ca^2+^, 2 mM Ca^2+^ and 5 mM Ca^2+^) and calcium channel blockers (25 μm SKF 96365 hydrochloride (Cat no. 1147, Tocris) and 50 μm 2-APB (D9754, Sigma)) for 24 h. After treatment, the medium was aspirated from each dish. Cells were washed with 1×PBS and were harvested by trypsinization. One million MSCs were fixed in 70% ethanol for 24 h, rinsed in 1×PBS, resuspended in 100 μl 1×PBS. Cells were incubated with 100 μg/ml RNase A (Cat no. AM2286, Invitrogen) at 37 °C for 1 h and then treated with 10 μg/μl PI (Cat no. P3566, Invitrogen) on ice for 15 min in the dark. About 20,000 events were captured by BD LSR-II using BD FACSDiva 8.0.1 software, and data were analyzed by Modfit software.

### Cell viability

Cell viability was determined by 3-(4,5-dimethyl thiazolyl-2)−2,5-diphenyltetrazolium bromide, MTT (Cat no. 475989, Calbiochem) assay. Briefly, cells (2 × 10^4^ cells/cm^2^) were seeded in 96-well plates and were incubated for 24 h at 37 °C in a 5% CO_2_ humidified incubator. After 24 h, the medium was aspirated from each well and a new medium containing different calcium conc. (0 mM Ca^2+^, 2 mM Ca^2+^, and 5 mM Ca^2+^) and calcium channel blockers (25 μm SKF 96365 and 50 μm 2-APB) were added to the wells, and cells were grown for 1 and 3 days. After day 1 and day 3, the medium was aspirated from each well, and 100 μl media containing 20 μl MTT reagent (5 mg/ml MTT in 1×PBS) was added to each well, and incubated in a CO_2_ humidified incubator for 4 h. The medium was removed from each well, and purple bluish formazan crystals were dissolved in 100 μl of acid alcohol (0.04 N HCl in isopropanol) for 15 min at RT. The absorbance was measured in a Synergy™ HTX Multi-Mode Microplate Reader at 570 and 630 nm.

### Colony-forming unit‑fibroblasts (CFU‑F) assay

The stem cell potential of the MSCs was analyzed by the CFU-F assay. Briefly, stem cells (2 × 10^6^ cells/well) were seeded in 6-well plates and incubated for 24 h at 37 °C in a 5% CO_2_ humidified incubator. After 1 day, the medium was aspirated from each well, and cells were treated with different calcium conc. (0 mM Ca^2+^, 2 mM Ca^2+^, and 5 mM Ca^2+^) and calcium channel blockers (25 μm SKF 96365 and 50 μm 2-APB) for 24 h. After treatment, the medium was aspirated from each well, and a new medium was added to the cells. On day 6th, the medium was aspirated from each well, and cells were fixed and stained with 1 ml crystal violet (CV) solution (Cat no. V5265, Sigma). The stain was aspirated, and cells were washed with 1×PBS. Then, 500 μl acetic acid (10%) was added to each well and incubated for 30 min on an RT shaker. CV solution was collected from each well, and absorbance was measured in a Synergy™ HTX Multi-Mode Microplate Reader at 550 and 590 nm.

### Calcium imaging

Calcium imaging was performed using Fura-2-AM (Cat no. 344905, Calbiochem), a fluorescence Molecular Probes as described earlier^27^. In brief, cells were cultured and then incubated with 2 μM fura-2-AM for 30 min. Media was removed and washed three times with Ca^2+^ free Standard External Solution, SES (10 mM HEPES, 120 mM NaCl, 5.4 mM KCl, 1 mM MgCl_2_, 10 mM glucose, pH7.4). The fluorescence measurements were achieved using the fluorescence intensity of Fura-2-loaded cells, captured by Olympus XL70 inverted microscope furnished with an Olympus 340 (1.3 NA) objective and a CCD camera-based imaging system (Compix, Lake Oswego, USA). Several images of cells were collected at excitation 340 nm and excitation at 380 nm wavelength. Images were processed using Phase Contrast X-ray Imaging, PCI software (Compix, Inc., Cranberry Township, PA, USA). The ratio (F340/F380) of 20–50 cells was acquired, and fluorescence traces were prepared from three individual experiments.

### Electrophysiology

Patch-clamp measurements were done as previously described^27^. In brief, very low cell density of cells was seeded on a coverslip, and coverslips with cells were transferred to the recording chamber. Coverslip was perfused with external Ringer’s solution (145 mM NaCl, 5 mM KCl, 1 mM MgCl_2_, 1 mM CaCl_2_, 10 mM HEPES, 10 mM glucose, pH 7.4 with NaOH base), and whole-cell currents were recorded by Axopatch 200B amplifier (Molecular Devices, LLC, Sunnyvale, CA, USA) in a tight-seal whole-cell configuration at RT. The patch pipette was filled with the standard intracellular solution (150 mM cesium methanesulfonate, 8 mM NaCl, 10 mM HEPES, 10 mM EGTA, pH 7.2 with CsOH base) and after filling resistances in the pipette was between 3 and 5 MΏ. At 0 mV holding potential, voltage ramps ranging from −80 mV to +80 mV were delivered to the cells for 100 ms at 2 s intervals. Currents were recorded (at 2 kHz) and digitized (at 5–8 kHz). Data acquisition and analysis were made by pClamp 10.1 software (Molecular Devices, LLC, Sunnyvale, CA, USA). Basal leak current was subtracted from the final current, and average currents from 8–10 cells in each condition were plotted.

### Protein isolation western blotting

Cells were harvested by trypsinization, centrifuged at 500 × *g*, 5 min, at 4 °C, and discarded supernatant. Cell pellets were lysed by sonication and RIPA lysis and Extraction buffer (Cat no. 89900, Thermo Scientific) containing complete™ protease inhibitors (Cat no. 05892970001, Roche) and phosphatase inhibitors (Cat no. 04906845001, Roche). Proteins were extracted by centrifugation of samples for 20 min at max speed at 4 °C. The concentration of proteins was quantified by Bio-Rad protein assay dye (Cat no. 5000006, Bio-Rad) according to the manufacturer’s instructions. Western blotting was performed as previously described^44^. In brief, Protein samples, 25 µg, were mixed with NuPAGE LDS sample buffer (Cat no. NP0007) & NuPAGE sample reducing agent (Cat no. NP0009) and boiled at 95 °C for 5 min. Then, samples were cooled and loaded into the wells of NuPAGE 4–12% mini protein gel (Cat no. NP0335BOX, Thermo Scientific), submerging in MOPS SDS running buffer (Cat no. NP0001, Thermo Scientific). Proteins were transferred from gel to iBlot transfer nitrocellulose membrane (Cat no. IB23002, Thermo Scientific). Blots were rinsed in water and stained with Ponceau S solution (Cat no. P7170, Sigma) to check the transfer quality. Ponceau S staining was removed by washing three times with PBST. Membranes were blocked with a 5% blotting-grade blocker (Cat no. 1706404, Bio-Rad) for 1 h at RT. Membranes were rinsed with PBST and incubated with primary antibodies [Orai1 (Cat no. MA5-15776, Invitrogen), STIM1 (Cat no. ab106531, Abcam), TRPC1 (Cat no. ab192031, Abcam) and β-Actin (Cat no. 4970, Cell signaling), with a dilution of 1:1000] overnight at 4 °C. Blots were rinsed three times with PBST and incubated with secondary antibody conjugated with HRP (Cat no. 32460, Invitrogen) with a dilution of 1:10,000 for 1 h at RT. All blots were from the same experiment and were processed in parallel, and according to the manufacturer’s recommendation, membrane blots were rinsed three times with PBST and developed by Clarity MaxTM western ECL substrate (Cat no.1705062, Bio-Rad). Chemiluminescent signals were captured using CCD camera-based ChemiDocTM XRS+ imager. Western blots were quantified by Image LabTM software^15,27,44^.

### Immunofluorescence

Intracellular localization of proteins was studied in the MSCs by immunofluorescence. Ten thousand cells were seeded on the glass bottom dish and incubated in the CO_2_ humidified incubator for 1 day at 37 °C. Cells were washed with 1 ml 1×PBS, fixed with 2 ml, 4% paraformaldehyde (PFA) for 30 min at RT followed by PFA was drained off, and cells were washed with 2 ml 1×PBS. Cells were then incubated in 100 mM glycine/PBS for 20 min at RT followed by glycine was aspirated, and cells were washed with 1×PBS three times. Cells were permeabilized with cold methanol (−70 °C) for 5 min. Then methanol was aspirated, and cells were washed with 1×PBS three times. Blocking was done with 5% donkey serum in 1×PBS/0.5% BSA for 20 min with shaking at RT followed by cells being washed with 1×PBS. After this, cells were incubated with primary antibodies NF-kB (Cat no. 3033, Cell Signaling), ERK (Cat no. 4695, Cell Signaling), JNK (Cat no. 9258, Cell Signaling), and β-Actin (Cat no. 4970, Cell signaling) at 1:250 dilutions as per recommendation for overnight at 4 °C. Unbound primary antibodies were washed out during washing with 2 ml PBST. Then cells were stained with PE-conjugated IgG secondary antibody (Cat no. ab7007) at 1:500 dilution as per recommendation for 1 h at RT. Unbound secondary antibodies were drained off by washing three times with 2 ml PBST. Then Vectasheild with DAPI (Vector Labs H-1200) was applied to the cells and mounted. Images were captured by a Leica fluorescence confocal microscope.

### Animal and development of the mouse model for salivary gland regeneration

Male C57BL/6J mice from Jackson Laboratories (6–8 weeks old) were used for all experiments. All mice were housed and maintained in the animal house facility of the University of Texas health sciences center, UTHSCSA, where standard conditions of housing, feed, water, temperature (25 °C), humidity, and 12 h:12 h light: dark cycle was maintained. The institutional IACUC committee approved all animal protocols, and then all experiments were performed as per the institutional guidelines on animal care by UTHSCSA. For radiation exposures, the mice were staged and positioned to expose the neck region with the help of CT guidance and infrared tracking in an SARRP-200 225 kVp multi-modality image-guided X-ray platform (Xstrahl Inc., Georgia) under continuous anesthesia and airflow. The X-ray beam was collimated to precisely expose the salivary gland region. According to previously published literature, a single dose of 15 Gy X-ray radiation was given to damage the salivary glands. Freshly isolated MSCs (1 × 10^6^) grown in either 2 mM calcium of 0.5 mm calcium were transplanted into the irradiated mice through tail vein injection^45^. Multiple transplantations were performed on day 0, day 4, and day 8. After the eighth day of the last transplantation, mice (control, irradiated, and transplanted mice) saliva secretion was measured. Mice were sacrificed by cervical dislocation and tissues were collected for histological examination.

### Histological studies and Immunohistochemistry

Salivary glands were aseptically harvested, washed with 1×PBS, and fixed in 10% formalin until used. The fixed glands were dehydrated in ethanol (30% for 30 min, 50% for 30 min, 70% for 30 min, 95% for 1 h, 100% for 1 h at 4 °C) and incubated in xylene (15 min at RT). Finally, the properly oriented salivary glands were immersed in pre-melted paraffin wax for 3 h at 60 °C. The paraffin blocks, embedded in metal blocks, were cut into 5-μm-thick sections by microtome. Tissue sections were spread on prewarmed (37 °C) water, mounted on gelatin-coated slides, and stored at RT. The tissue sections were deparaffinized with xylene (2 min), hydrated (with 100%, 95%, 70%, 50%, and 30% for 5 min at RT), and stained with Hematoxylin (1 min). The excess stain was removed by acid alcohol, subsequently dehydrated through ethanol, and stained with eosin (30 s) followed by dehydration with 95% and 100% ethanol, and finally treated with xylene and mounted with DPX. Images of salivary gland sections were taken at different magnifications. For Immunohistochemistry, paraffin-embedded tissue sections were used for immunostaining. Recovery of salivary glands was analyzed by AQ5 (Cat no. PA5-36529), Na^+^/K^+^-ATPase (Cat no. sc-71638), and TRPC1 expression and transplantation efficiency of MSCs was measured by CD44 occurrence in irradiated and transplanted mice. Salivary gland (5 μm) sections were deparaffinized and rehydrated through ethanol with 100%, 95%, 70%, 50%, and 30% for 5 min at RT followed by the immunofluorescence protocol was used. Images were captured by a Leica fluorescence confocal microscope.

### Statistical analysis

All experiments were replicated, and experimental data values are expressed as the mean ± standard error of the mean (SEM). One‐way ANOVA or Student *t*-test were performed for significant differences among the experimental groups. *p*-value > 0.05 was termed not significant (and labeled as NS). *p* < 0.05 or higher was considered statistically significant and is denoted in the figure legends.

### Reporting summary

Further information on research design is available in the Nature Research Reporting Summary linked to this article.

## Supplementary information

Supplementary Information

Reporting Summary

## Data Availability

The data included in this manuscript are available from the corresponding author upon request.
