# Supplementary material for: Differential activation of Ca2+ influx channels modulate stem cell potency, their proliferation/viability and tissue regeneration
Source: NPJ Regen Med. 2021 Oct 20;6:67. doi: 10.1038/s41536-021-00180-w (PMC8528841; doi:10.1038/s41536-021-00180-w)
Supplement: Supplementary file 1 — Supplementary Information [file 41536_2021_180_MOESM1_ESM.pdf]

SUPPLEMENTARY FIGURE LEGENDS:

Supplementary Figure 1: Characterization of mesenchymal stem cells **a, b** Immunophenotypic characterization of MSCs using flow cytometer showing the absolute counts of MSC positive and negative markers. **c, d** Microscopic images showing MSCs differentiation into adipocytes and osteocytes lineages. Lipid droplets (oil red staining) and mineral deposition (Van Kossa staining) was used to evaluate adipocyte and osteocyte differentiation respectively.

Supplementary Figure 2: Expression of PCNA after different calcium doses and upon treatment with various calcium channel blockers. **a-c, f** displaying (histograms and bar graph) the percentage of cells showing PCNA expression after treatment of 0 mM Ca^{2+}, 2 mM Ca^{2+}, 5 mM Ca^{2+}, respectively. **d-e, g** shows (histograms and graph) of the percentage of cells showing PCNA expression after 50 µM 2-APB or 25 µM SKF 96365 treatment, respectively. **h-i** showing intracellular calcium level after treatment with different calcium doses (0 mM Ca^{2+}, 2 mM Ca^{2+}, 5 mM $Ca^{2+)}$ and calcium channels blockers (50 µM 2-APB and 25 µM SKF 96365), respectively. **j,** shows western blots showing downregulation of individual proteins in cells transfected with a particular siRNA as labelled. Actin was used as control.

Supplementary Figure 3: Transcription factor expression and food and water intake in mice. **a** showing immunofluorescence of pERK, pJNK, and β-Actin after Tg treatment. **b** showing quantification of pNFkB after various treatment as labeled, $^*p \leq 0.001$ (Student's t test). **c** Image showing the irradiated salivary gland area. **d, e** graphs represent body weight changes, amount of water intake, food consumed by control mice or mice after radiation and transplantation, respectively. **f**, shows quantification (n=4) of CD44 in salivary gland under various conditions. $^*p \leq 0.001$ (Student's t test).

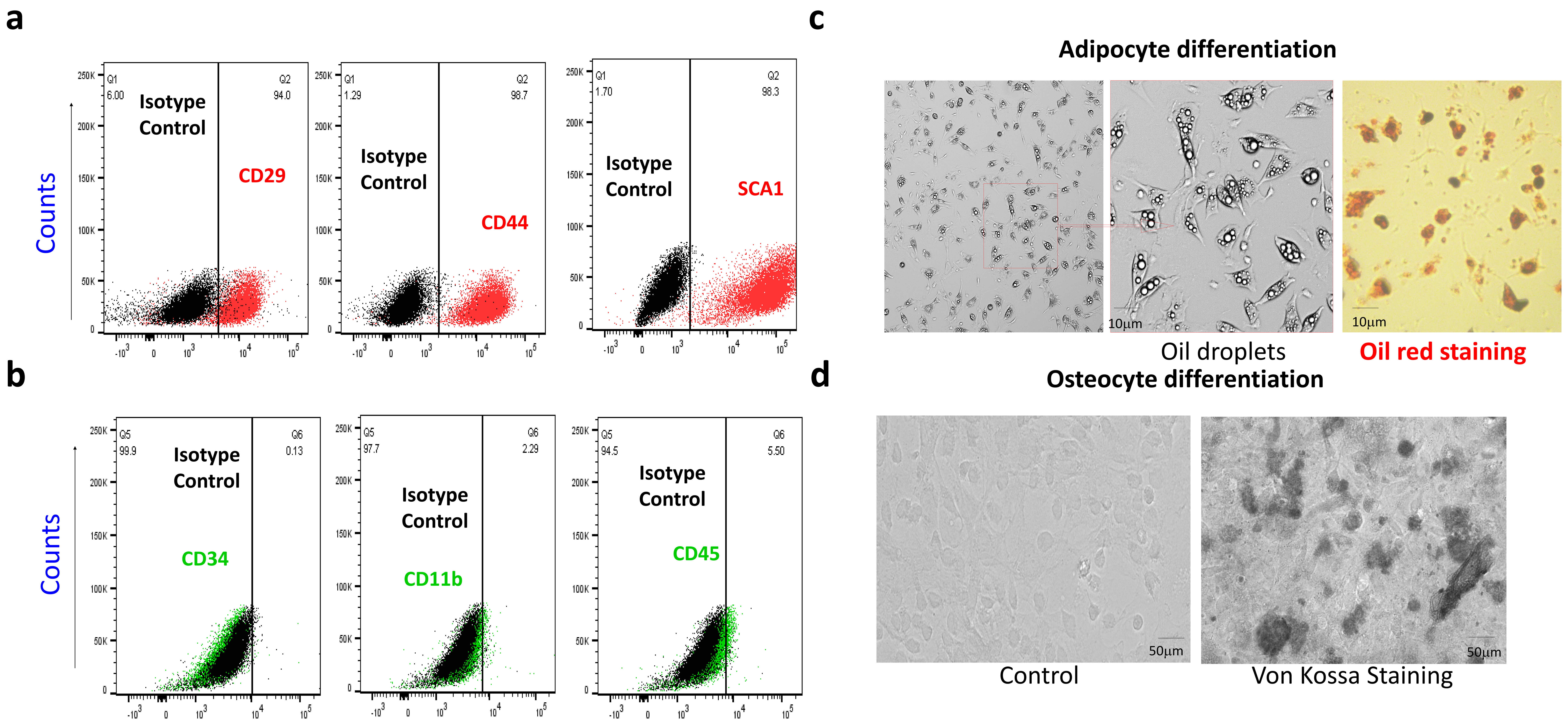
a
b
c
d
Counts
Isotype Control
CD29
CD44
SCA1
Q1
6.00
Q2
94.0
1.29
98.7
1.70
98.3
Counts
CD34
CD11b
CD45
Q5
99.9
Q6
0.13
97.7
2.29
94.5
5.50
Adipocyte differentiation
10µm
Oil droplets
Oil red staining
Osteocyte differentiation
50µm
Control
Von Kossa Staining

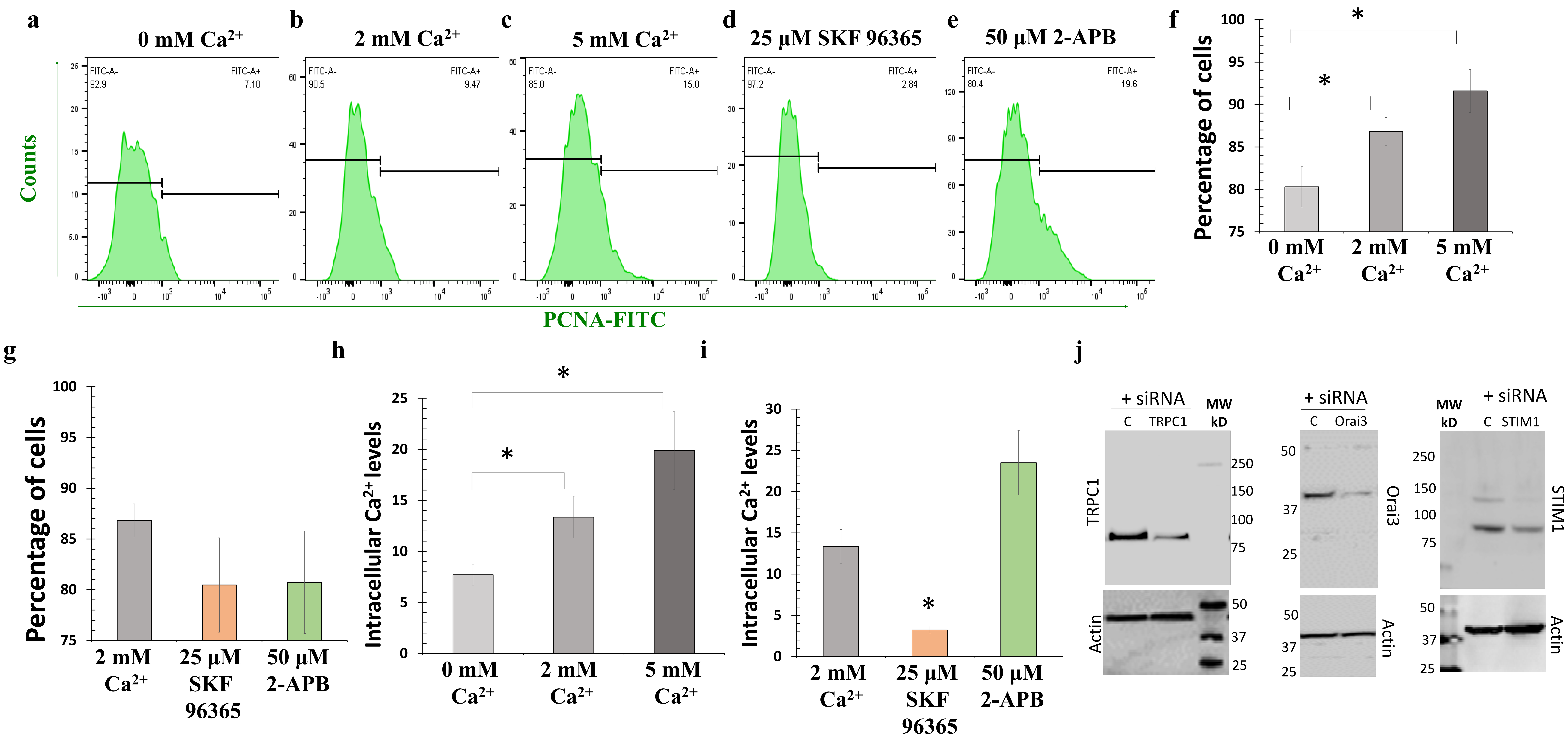

a
0 mM Ca2+
b
2 mM Ca2+
c
5 mM Ca2+
d
25 µM SKF 96365
e
50 µM 2-APB
f
Counts
PCNA-FITC
Percentage of cells
0 mM Ca2+
2 mM Ca2+
5 mM Ca2+
g
Percentage of cells
2 mM Ca2+
25 µM SKF 96365
50 µM 2-APB
h
Intracellular Ca2+ levels
0 mM Ca2+
2 mM Ca2+
5 mM Ca2+
i
Intracellular Ca2+ levels
2 mM Ca2+
25 µM SKF 96365
50 µM 2-APB
j
+ siRNA
C
TRPC1
MW kD
TRPC1
Actin
Orai3
STIM1

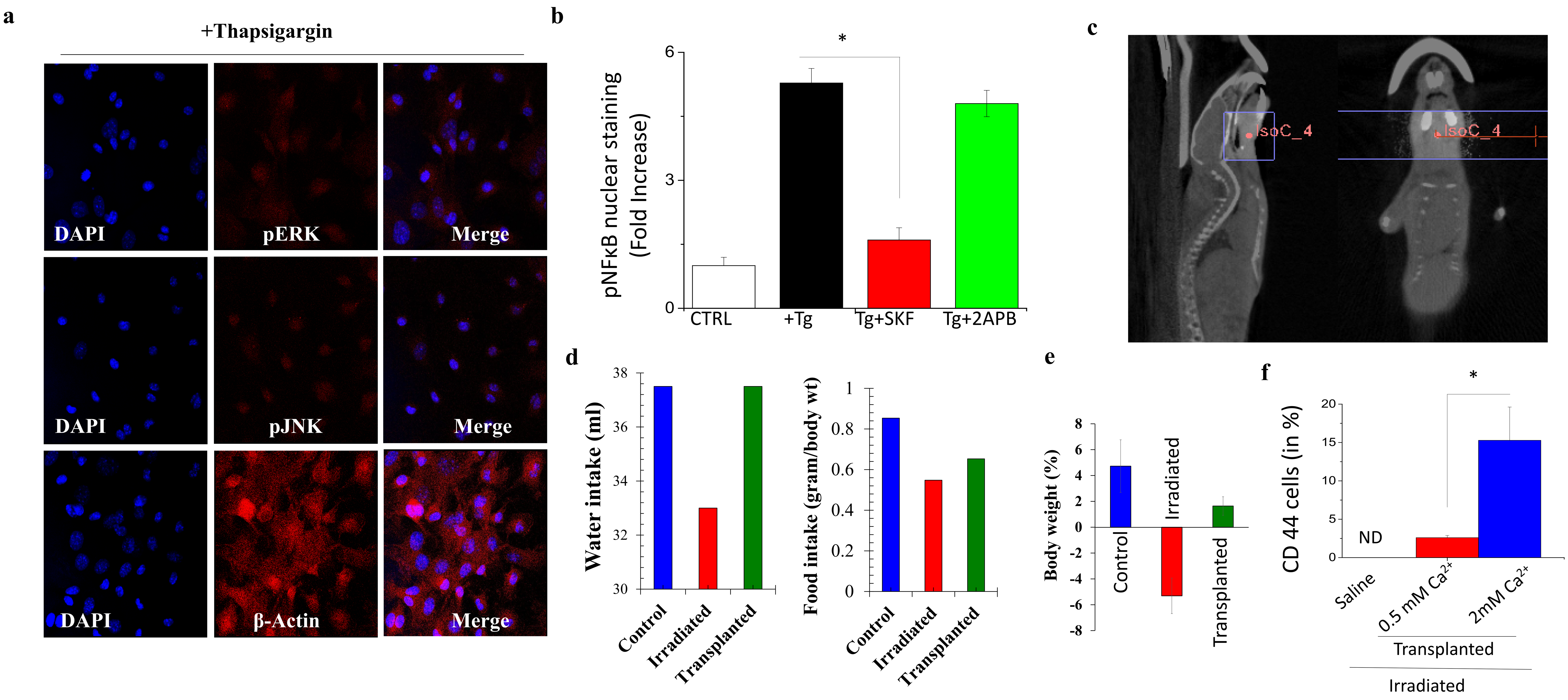
a
+Thapsigargin
DAPI
pERK
Merge
DAPI
pJNK
Merge
DAPI
β-Actin
Merge
b
pNFκB nuclear staining (Fold Increase)
CTRL
+Tg
Tg+SKF
Tg+2APB
c
IsoC_4
IsoC_4
d
Water intake (ml)
Food intake (gram/body wt)
Control
Irradiated
Transplanted
e
Body weight (%)
Control
Irradiated
Transplanted
f
CD 44 cells (in %)
ND
Saline
0.5 mM Ca2+
2mM Ca2+
Transplanted
Irradiated
