# Supplementary material for: Differential activation of Ca2+ influx channels modulate stem cell potency, their proliferation/viability and tissue regeneration
Source: NPJ Regen Med. 2021 Oct 20;6:67. doi: 10.1038/s41536-021-00180-w (PMC8528841; doi:10.1038/s41536-021-00180-w)
Supplement: Supplementary file 2 — Reporting Summary [file 41536_2021_180_MOESM2_ESM.pdf]

nature portfolio

Corresponding author(s): Brij B Singh

Last updated by author(s): Jul 13, 2021

Reporting Summary

Nature Portfolio wishes to improve the reproducibility of the work that we publish. This form provides structure for consistency and transparency in reporting. For further information on Nature Portfolio policies, see our Editorial Policies and the Editorial Policy Checklist.

Statistics

For all statistical analyses, confirm that the following items are present in the figure legend, table legend, main text, or Methods section.

n/a	Confirmed	
☐	☒	The exact sample size (*n*) for each experimental group/condition, given as a discrete number and unit of measurement
☐	☒	A statement on whether measurements were taken from distinct samples or whether the same sample was measured repeatedly
☐	☒	The statistical test(s) used AND whether they are one- or two-sided<br>*Only common tests should be described solely by name; describe more complex techniques in the Methods section.*
☒	☐	A description of all covariates tested
☐	☒	A description of any assumptions or corrections, such as tests of normality and adjustment for multiple comparisons
☐	☒	A full description of the statistical parameters including central tendency (e.g. means) or other basic estimates (e.g. regression coefficient) AND variation (e.g. standard deviation) or associated estimates of uncertainty (e.g. confidence intervals)
☐	☒	For null hypothesis testing, the test statistic (e.g. *F*, *t*, *r*) with confidence intervals, effect sizes, degrees of freedom and *P* value noted<br>*Give P values as exact values whenever suitable.*
☒	☐	For Bayesian analysis, information on the choice of priors and Markov chain Monte Carlo settings
☒	☐	For hierarchical and complex designs, identification of the appropriate level for tests and full reporting of outcomes
☒	☐	Estimates of effect sizes (e.g. Cohen's *d*, Pearson's *r*), indicating how they were calculated

Our web collection on statistics for biologists contains articles on many of the points above.

Software and code

Policy information about availability of computer code

Data collection	GraphPad Prism 8.0 (GraphPad Software, Inc. San Diego, CA, USA); OriginLab, (Originlab Corporation) Northampton, MA, USA
Data analysis	GraphPad Prism 8.0 (GraphPad Software, Inc. San Diego, CA, USA); OriginLab, (Originlab Corporation) Northampton, MA, USA

For manuscripts utilizing custom algorithms or software that are central to the research but not yet described in published literature, software must be made available to editors and reviewers. We strongly encourage code deposition in a community repository (e.g. GitHub). See the Nature Portfolio guidelines for submitting code & software for further information.

Data

Policy information about availability of data

All manuscripts must include a data availability statement. This statement should provide the following information, where applicable:

- Accession codes, unique identifiers, or web links for publicly available datasets
- A description of any restrictions on data availability
- For clinical datasets or third party data, please ensure that the statement adheres to our policy

The data presented in this manuscript are available by the corresponding author on reasonable request.

Field-specific reporting

Please select the one below that is the best fit for your research. If you are not sure, read the appropriate sections before making your selection.

☒ Life sciences ☐ Behavioural & social sciences ☐ Ecological, evolutionary & environmental sciences

For a reference copy of the document with all sections, see nature.com/documents/nr-reporting-summary-flat.pdf

Life sciences study design

All studies must disclose on these points even when the disclosure is negative.

Sample size	Sample size calculations were not performed. Three or more independent experiments with biological replicates were carried out for each experiment.
Data exclusions	No exclusions
Replication	3 or more independent replicates are included in the results.
Randomization	For in vivo studies all animals were randomly selected and divided into 3 groups as described in the text.
Blinding	Animals were blinded for saliva secretion as well as samples for confocal or FACS were assigned a randomized file names for the assessor.

Reporting for specific materials, systems and methods

We require information from authors about some types of materials, experimental systems and methods used in many studies. Here, indicate whether each material, system or method listed is relevant to your study. If you are not sure if a list item applies to your research, read the appropriate section before selecting a response.

Materials & experimental systems

n/a	Involved in the study	
☐	☒	Antibodies
☒	☐	Eukaryotic cell lines
☒	☐	Palaeontology and archaeology
☐	☒	Animals and other organisms
☒	☐	Human research participants
☒	☐	Clinical data
☒	☐	Dual use research of concern

Methods

n/a	Involved in the study	
☒	☐	ChIP-seq
☐	☒	Flow cytometry
☒	☐	MRI-based neuroimaging

Antibodies

Antibodies used	CD29 (Cat no. 12-0291-82), CD44 (Cat no. 12-0441-82), Ly-6A/E (Sca-1) (Cat no. 12-5981-82), CD11b (Cat no. 11-0112-82), CD45 (Cat no. 11 0451-82), CD34 (Cat no. 11-0341-82)] ; Orai1 (Cat no. MA5-15776, Invitrogen), STIM1 (Cat no. ab106531, Abcam), TRPC1 (Cat no. ab192031, Abcam) pNFkB (Cat no. 3033, Cell Signaling), ERK (Cat no. 4695, Cell Signaling), JNK (Cat no. 9258, Cell Signaling)and β-Actin (Cat no. 4970, Cell signaling),
Validation	All the antibodies used were from commercial sources which has been validated for their individual use and have been included in multiple publications. In addition isotype controls were used for all antibodies used for FACS analysis. The catalogue numbers are included by teh antibodies used and all relevant data including validation is on their websites.

Animals and other organisms

Policy information about studies involving animals; ARRIVE guidelines recommended for reporting animal research

Laboratory animals	C57BL6 mice were used for the study
Wild animals	None used
Field-collected samples	N/A
Ethics oversight	All animal protocols were approved by the Institutional Animal Care and Use Committee oat the University of Texas Health San Antonio.

Note that full information on the approval of the study protocol must also be provided in the manuscript.

Flow Cytometry

Plots

Confirm that:

☒ The axis labels state the marker and fluorochrome used (e.g. CD4-FITC).

☒ The axis scales are clearly visible. Include numbers along axes only for bottom left plot of group (a 'group' is an analysis of identical markers).

☒ All plots are contour plots with outliers or pseudocolor plots.

☒ A numerical value for number of cells or percentage (with statistics) is provided.

Methodology

Sample preparation	The Methods section details how the samples were prepared
Instrument	BD LSR II flow cytometer (BD Biosciences)
Software	FlowJo software v10.7 (Becton, Dickinson and Company)
Cell population abundance	N/A.
Gating strategy	FSC between 10k to 200k, and SSC between 10k to 200k were used to gate the single cells. Cells with isotype controls were used as negative control for gating.

☒ Tick this box to confirm that a figure exemplifying the gating strategy is provided in the Supplementary Information.
